# {4,4′,6,6′-Tetrachloro-2,2′-[2,2-dimethyl­propane-1,3-diylbis(nitrilo­methanylyl­idene)]diphenolato}dioxidomolyb­denum(VI)

**DOI:** 10.1107/S160053681203807X

**Published:** 2012-09-08

**Authors:** Hadi Kargar, Maciej Kubicki

**Affiliations:** a Department of Chemistry, Payame Noor University, PO Box 19395-3697 Tehran, IR of IRAN; b Department of Chemistry, Adam Mickiewicz University, Grunwaldzka 6, 60-780 Poznán, Poland

## Abstract

The asymmetric unit of the title compound, [Mo(C_19_H_16_Cl_4_N_2_O_2_)O_2_], comprises two independent mol­ecules (*A* and *B*). The geometry around the Mo^VI^ atom is distorted octa­hedral in each complex mol­ecule, supported by two oxide O atoms and the N_2_O_2_ donor atoms of the coordinating ligand. The dihedral angle between the benzene rings is 74.96 (11) Å for mol­ecule *A* and 76.05 (11) Å for mol­ecule *B*. In the crystal, the *B* mol­ecules are linked by pairs of C—H⋯Cl hydrogen bonds, forming inversion dimers. The crystal structure is further stabilized by C—H⋯π inter­actions. An inter­esting feature of the crystal structure is a Cl⋯Cl contact [3.3748 (18) Å], which is shorter than the sum of the van der Waals radii of Cl atoms (3.50 Å).

## Related literature

For the importance of molybdenum in molybdoenzymes and in coordination chemistry and catalysis, see, for example: Majumdar & Sarkar (2011 ▶); Enemark *et al.* (2004 ▶); Mancka & Plass (2007 ▶). For background to Schiff base ligands, their complexes with MoO_2_, and related structures, see, for example: Kia & Fun (2009 ▶); Kargar & Kia (2011 ▶); Abbasi *et al.* (2008 ▶); Monadi *et al.* (2009 ▶). For standard values of bond lengths, see: Allen *et al.* (1987 ▶). For van der Waals radii, see: Bondi (1964 ▶).
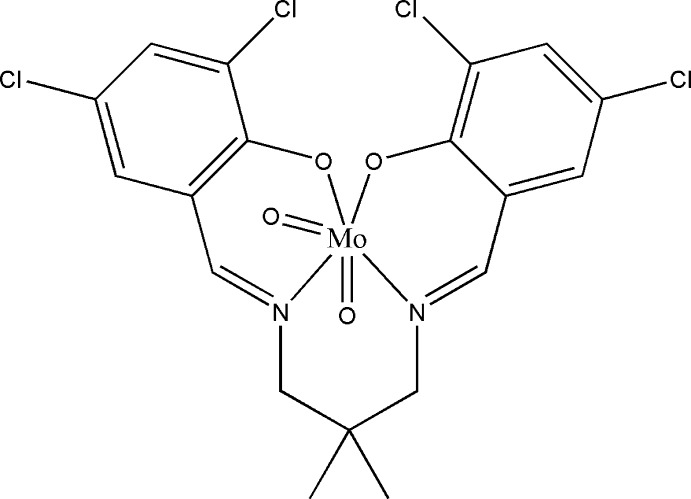


## Experimental

### 

#### Crystal data

[Mo(C_19_H_16_Cl_4_N_2_O_2_)O_2_]
*M* *_r_* = 574.08
Monoclinic, 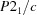

*a* = 12.840 (5) Å
*b* = 15.457 (5) Å
*c* = 22.173 (5) Å
β = 102.397 (5)°
*V* = 4298 (2) Å^3^
*Z* = 8
Cu *K*α radiation
μ = 9.84 mm^−1^
*T* = 296 K
0.42 × 0.21 × 0.11 mm

#### Data collection

Agilent Super Nova Atlas CCD area-detector diffractometer
Absorption correction: multi-scan (*CrysAlis PRO*; Agilent, 2011 ▶) *T*_min_ = 0.104, *T*_max_ = 0.411
67900 measured reflections
8626 independent reflections
8373 reflections with *I* > 2σ(*I*)
*R*_int_ = 0.049

#### Refinement

*R*[*F* ^2^ > 2σ(*F* ^2^)] = 0.027
*wR*(*F* ^2^) = 0.079
*S* = 1.07
8626 reflections
541 parameters
H-atom parameters constrained
Δρ_max_ = 0.69 e Å^−3^
Δρ_min_ = −0.71 e Å^−3^

### 

Data collection: *CrysAlis PRO* (Agilent, 2011 ▶); cell refinement: *CrysAlis PRO*; data reduction: *CrysAlis PRO*; program(s) used to solve structure: *SIR92* (Altomare *et al.*, 1993 ▶); program(s) used to refine structure: *SHELXL97* (Sheldrick, 2008 ▶); molecular graphics: *SHELXTL* (Sheldrick, 2008 ▶); software used to prepare material for publication: *SHELXTL* and *PLATON* (Spek, 2009 ▶).

## Supplementary Material

Crystal structure: contains datablock(s) global, I. DOI: 10.1107/S160053681203807X/su2496sup1.cif

Structure factors: contains datablock(s) I. DOI: 10.1107/S160053681203807X/su2496Isup2.hkl

Additional supplementary materials:  crystallographic information; 3D view; checkCIF report

## Figures and Tables

**Table 1:** Hydrogen-bond geometry (Å, °) *Cg1* is the centroid of the C20–C25 ring in mol­ecule *B* and *Cg2* is the centroid of the C12–C17 ring in mol­ecule *A*.

*D*—H⋯*A*	*D*—H	H⋯*A*	*D*⋯*A*	*D*—H⋯*A*
C38—H38*B*⋯Cl8^i^	0.96	2.82	3.767 (3)	168
C10—H10*B*⋯*Cg*1^ii^	0.97	2.66	3.433 (3)	136
C27—H27*A*⋯*Cg*2^iii^	0.97	2.55	3.363 (3)	141

Symmetry codes: (i) 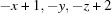; (ii) 
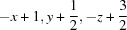
; (iii) 
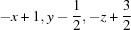
.

**Fig. 1.**
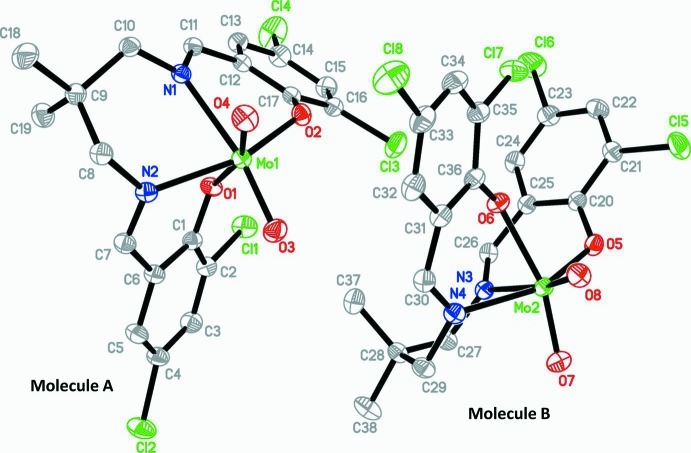
The molecular structure of the title compound, showing the atom numbering. Displacement ellipsoids are drawn at the 40% probability level.The H atoms have been omitted for clarity.

**Fig. 2.**
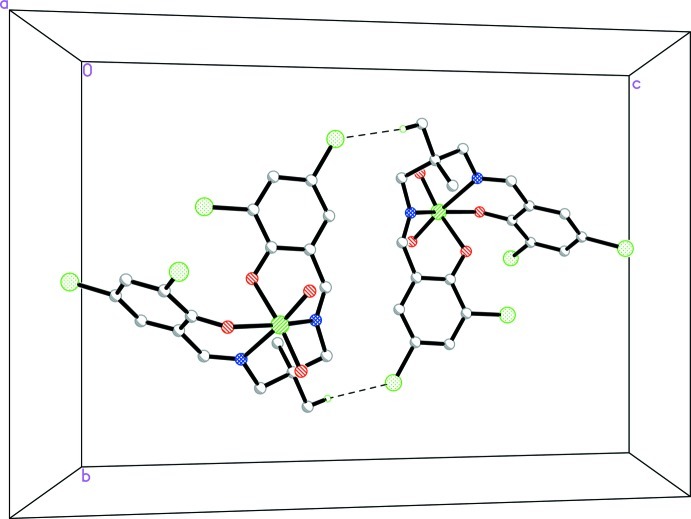
A partial view along the a axis of the crystal packing of the title complex, showing the linking of B molecules through intermolecular C—H···Cl interactions (dashed lines; only the H atoms involved in these interactions are shown; see Table 1 for details).

**Fig. 3.**
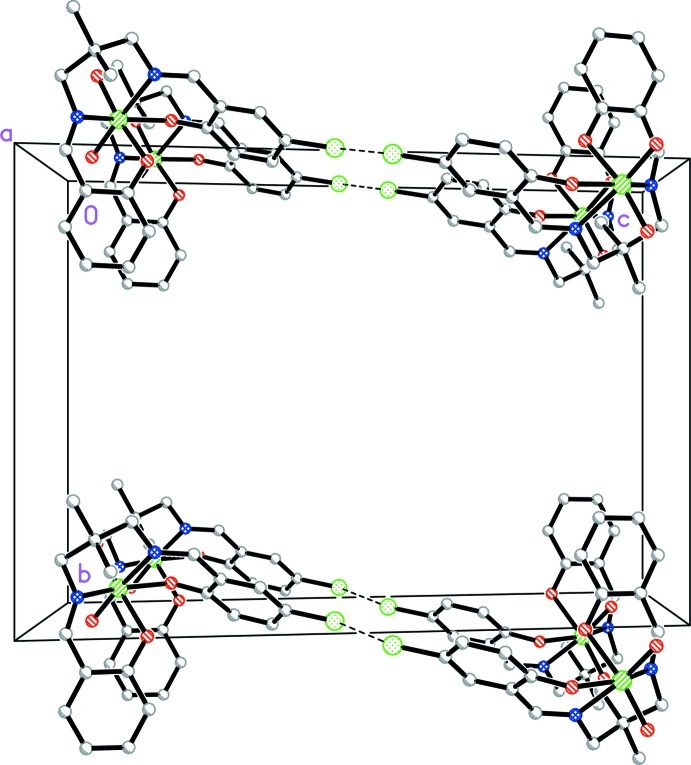
A partial view along the a axis of the crystal packing of the title complex showing the linking of the molecules through intermolecular Cl···Cl interactions (dashed lines). Only the Cl atoms involved in these interactions are shown.

## supplementary crystallographic information

### Comment

The element molybdenum is unique among metals due to its varied roles, for instance in the form of bio-catalysts as found in the enzymatic reactions in several natural molybdoproteins (Majumdar & Sarkar, 2011). The coordination chemistry of molybdenum(VI) has attracted considerable attention due to its biological importance (Enemark *et al.*, 2004) and to their application in various catalytic oxidation reactions (Mancka & Plass, 2007). In continuation of our work on the crystal structure of Schiff base ligands from different substituted salicylaldehyde and amines and their complexes (Kargar & Kia, 2011; Kia & Fun, 2009) we synthesized and determined the X-ray structure of the title compound.

The asymmetric unit of the title compound comprises two crystallographically independent molecules, A and B, as shown in Fig. 1. Each Mo^VI^ centre is coordinated by two oxide O-atoms and by two O and two N atoms of the tetradentate Schiff base ligand in a distorted octahedral configuration. The dihedral angles between the benzene rings in the the two compounds are 74.96 (11) for rings C1-C6 and C12-C17 in A, and 76.05 (11) Å for rings C20-C25 and C31-C36 in B. The bond lengths and angles are within the normal ranges (Allen *et al.*, 1987), and are comparable to those reported for similar structures (Abbasi *et al.*, 2008; Monadi *et al.*, 2009). The Mo1—N1 and Mo2—N3 bond lengths [2.3347 (17) and 2.3391 (17) Å, respectively] are *trans* to the terminal oxo groups and are significantly longer than the Mo1—N2 and Mo2—N4 bond lengths [2.1547 (18) and 2.1621 (18) Å, respectively]. This can be attributed to the strong *trans* effect of the oxo group.

In the crystal, the B molecules are linked by a pair of C-H···Cl hydrogen bonds to form inversion dimers (Table 1 and Fig. 2). The crystal structure is further stabilized by intermolecular C—H···π interactions (Table 1). An interesting feature of the crystal structure is a Cl4···Cl4^i^ contact [3.3748 (18) Å; symmetry code: (i)-x, -y, -z+1; see Fig.3], which is shorter than the sum of the van der Waals radii of Cl atoms [3.50 Å; Bondi 1964].

### Experimental

The title dioxidomolybdenum (VI) complex was prepared by mixing MoO_2_(acac)_2_ with the ligand bis(3,5-dichlorosalicylidene)-2,2-dime thyl-1,3-propandiamine, in a 1:1 molar ratio using 50 ml of methanol as solvent, followed by refluxing the solution for 2 h. The small reddish crystals that formed were filtered off and recrystallized from acetonitrile.

### Refinement

The H-atoms were included in calculated positions and treated as riding atoms: C—H = 0.93, 0.97 and 0.96 Å for CH, CH_2_ and CH_3_ H-atoms, respectively, with U_iso_ (H) = k × U_eq_(C), where k = 1.2 for CH, CH_2_ and 1.5 for CH_3_.

### Crystal data

**Table:**

[Mo(C_19_H_16_Cl_4_N_2_O_2_)O_2_]	*F*(000) = 2288
*M**_r_* = 574.08	*D*_x_ = 1.774 Mg m^−^^3^
Monoclinic, *P*2_1_/*c*	Cu *K*α radiation, λ = 1.54178 Å
Hall symbol: -P 2ybc	Cell parameters from 5865 reflections
*a* = 12.840 (5) Å	θ = 2.6–65.9°
*b* = 15.457 (5) Å	µ = 9.84 mm^−^^1^
*c* = 22.173 (5) Å	*T* = 296 K
β = 102.397 (5)°	Block, red
*V* = 4298 (2) Å^3^	0.42 × 0.21 × 0.11 mm
*Z* = 8	

### Data collection

**Table:**

Agilent Super Nova Atlas CCD area-detector diffractometer	8626 independent reflections
Radiation source: fine-focus sealed tube	8373 reflections with *I* > 2σ(*I*)
Graphite monochromator	*R*_int_ = 0.049
φ and ω scans	θ_max_ = 73.8°, θ_min_ = 3.5°
Absorption correction: multi-scan (*CrysAlis PRO*; Agilent, 2011)	*h* = −15→15
*T*_min_ = 0.104, *T*_max_ = 0.411	*k* = −17→19
67900 measured reflections	*l* = −27→27

### Refinement

**Table:**

Refinement on *F*^2^	Primary atom site location: structure-invariant direct methods
Least-squares matrix: full	Secondary atom site location: difference Fourier map
*R*[*F*^2^ > 2σ(*F*^2^)] = 0.027	Hydrogen site location: inferred from neighbouring sites
*wR*(*F*^2^) = 0.079	H-atom parameters constrained
*S* = 1.07	*w* = 1/[σ^2^(*F*_o_^2^) + (0.0456*P*)^2^ + 2.3531*P*] where *P* = (*F*_o_^2^ + 2*F*_c_^2^)/3
8626 reflections	(Δ/σ)_max_ = 0.004
541 parameters	Δρ_max_ = 0.69 e Å^−^^3^
0 restraints	Δρ_min_ = −0.71 e Å^−^^3^

### Special details

**Table:**

Geometry. All esds (except the esd in the dihedral angle between two l.s. planes) are estimated using the full covariance matrix. The cell esds are taken into account individually in the estimation of esds in distances, angles and torsion angles; correlations between esds in cell parameters are only used when they are defined by crystal symmetry. An approximate (isotropic) treatment of cell esds is used for estimating esds involving l.s. planes.
Refinement. Refinement of F^2^ against ALL reflections. The weighted R-factor wR and goodness of fit S are based on F^2^, conventional R-factors R are based on F, with F set to zero for negative F^2^. The threshold expression of F^2^ > 2sigma(F^2^) is used only for calculating R-factors(gt) etc. and is not relevant to the choice of reflections for refinement. R-factors based on F^2^ are statistically about twice as large as those based on F, and R- factors based on ALL data will be even larger.

### Fractional atomic coordinates and isotropic or equivalent isotropic displacement parameters (Å^2^)

**Table:**

	*x*	*y*	*z*	*U*_iso_*/*U*_eq_	
C1	0.17569 (16)	−0.08382 (12)	0.85123 (9)	0.0310 (4)	
C2	0.16664 (17)	−0.16416 (13)	0.82008 (10)	0.0346 (4)	
C3	0.14813 (18)	−0.23973 (14)	0.84863 (11)	0.0410 (5)	
H3A	0.1460	−0.2922	0.8279	0.049*	
C4	0.13259 (19)	−0.23703 (14)	0.90906 (11)	0.0413 (5)	
C5	0.13148 (19)	−0.15964 (14)	0.93932 (10)	0.0400 (5)	
H5A	0.1162	−0.1583	0.9784	0.048*	
C6	0.15359 (17)	−0.08231 (13)	0.91094 (10)	0.0337 (4)	
C7	0.13849 (17)	−0.00005 (14)	0.93867 (9)	0.0346 (4)	
H7A	0.0980	0.0005	0.9688	0.041*	
C8	0.13229 (18)	0.15290 (14)	0.94477 (9)	0.0346 (4)	
H8A	0.1901	0.1922	0.9616	0.042*	
H8B	0.0951	0.1395	0.9773	0.042*	
C9	0.05465 (17)	0.19803 (13)	0.89144 (9)	0.0341 (4)	
C10	0.11182 (17)	0.23127 (13)	0.84194 (9)	0.0337 (4)	
H10A	0.0601	0.2609	0.8104	0.040*	
H10B	0.1648	0.2735	0.8608	0.040*	
C11	0.14200 (16)	0.16007 (13)	0.75311 (9)	0.0309 (4)	
H11A	0.0876	0.1955	0.7322	0.037*	
C12	0.19467 (16)	0.10359 (13)	0.71609 (9)	0.0302 (4)	
C13	0.14309 (17)	0.08803 (14)	0.65515 (10)	0.0360 (4)	
H13A	0.0771	0.1131	0.6392	0.043*	
C14	0.19003 (19)	0.03533 (16)	0.61842 (10)	0.0419 (5)	
C15	0.28885 (19)	−0.00147 (15)	0.64065 (10)	0.0412 (5)	
H15A	0.3196	−0.0373	0.6156	0.049*	
C16	0.34122 (17)	0.01586 (14)	0.70061 (10)	0.0356 (4)	
C17	0.29588 (16)	0.06813 (13)	0.73944 (9)	0.0305 (4)	
C18	0.0123 (2)	0.27872 (17)	0.91895 (12)	0.0546 (6)	
H18A	−0.0366	0.3089	0.8871	0.082*	
H18B	0.0708	0.3161	0.9362	0.082*	
H18C	−0.0238	0.2615	0.9507	0.082*	
C19	−0.03728 (18)	0.13821 (17)	0.86320 (12)	0.0464 (5)	
H19A	−0.0846	0.1676	0.8301	0.070*	
H19B	−0.0755	0.1221	0.8942	0.070*	
H19C	−0.0097	0.0872	0.8474	0.070*	
C20	0.79437 (16)	−0.11005 (13)	0.73736 (9)	0.0311 (4)	
C21	0.84134 (17)	−0.05532 (14)	0.70059 (10)	0.0365 (4)	
C22	0.7898 (2)	−0.03316 (15)	0.64125 (10)	0.0420 (5)	
H22A	0.8217	0.0043	0.6178	0.050*	
C23	0.6901 (2)	−0.06753 (16)	0.61735 (10)	0.0433 (5)	
C24	0.64186 (18)	−0.12314 (15)	0.65155 (10)	0.0381 (5)	
H24A	0.5757	−0.1472	0.6343	0.046*	
C25	0.69258 (16)	−0.14329 (13)	0.71208 (9)	0.0320 (4)	
C26	0.63802 (16)	−0.20147 (13)	0.74708 (9)	0.0325 (4)	
H26A	0.5836	−0.2359	0.7249	0.039*	
C27	0.60340 (17)	−0.27598 (13)	0.83301 (9)	0.0344 (4)	
H27A	0.6549	−0.3197	0.8512	0.041*	
H27B	0.5519	−0.3035	0.8002	0.041*	
C28	0.54491 (17)	−0.24485 (14)	0.88253 (9)	0.0355 (4)	
C29	0.62282 (18)	−0.20500 (14)	0.93808 (9)	0.0376 (4)	
H29A	0.5852	−0.1936	0.9708	0.045*	
H29B	0.6792	−0.2462	0.9536	0.045*	
C30	0.63214 (17)	−0.05162 (15)	0.93682 (9)	0.0353 (4)	
H30A	0.5898	−0.0539	0.9660	0.042*	
C31	0.64975 (16)	0.03220 (14)	0.91217 (10)	0.0351 (4)	
C32	0.62645 (18)	0.10783 (16)	0.94214 (10)	0.0416 (5)	
H32A	0.6087	0.1044	0.9806	0.050*	
C33	0.63006 (19)	0.18659 (15)	0.91419 (12)	0.0456 (5)	
C34	0.64964 (19)	0.19296 (15)	0.85511 (11)	0.0436 (5)	
H34A	0.6496	0.2468	0.8363	0.052*	
C35	0.66914 (17)	0.11924 (14)	0.82456 (10)	0.0364 (4)	
C36	0.67446 (15)	0.03693 (13)	0.85286 (9)	0.0317 (4)	
C38	0.4984 (2)	−0.32662 (17)	0.90572 (12)	0.0532 (6)	
H38A	0.4492	−0.3533	0.8721	0.080*	
H38B	0.4619	−0.3114	0.9377	0.080*	
H38C	0.5551	−0.3663	0.9218	0.080*	
C37	0.45606 (18)	−0.18178 (17)	0.85567 (11)	0.0463 (5)	
H37A	0.4086	−0.2082	0.8212	0.070*	
H37B	0.4863	−0.1304	0.8421	0.070*	
H37C	0.4173	−0.1669	0.8867	0.070*	
Cl1	0.18472 (5)	−0.16526 (4)	0.74514 (2)	0.04789 (13)	
Cl2	0.11438 (6)	−0.33336 (4)	0.94560 (3)	0.05803 (16)	
Cl3	0.46752 (5)	−0.02498 (5)	0.72795 (3)	0.05550 (16)	
Cl4	0.12400 (7)	0.01570 (6)	0.54286 (3)	0.0760 (2)	
Cl5	0.96740 (5)	−0.01532 (5)	0.73031 (3)	0.05583 (15)	
Cl6	0.62383 (7)	−0.03848 (6)	0.54341 (3)	0.0740 (2)	
Cl7	0.69243 (5)	0.12454 (4)	0.75088 (3)	0.04899 (14)	
Cl8	0.60902 (7)	0.28117 (4)	0.95216 (4)	0.06755 (19)	
Mo1	0.299788 (11)	0.081572 (10)	0.872639 (6)	0.02889 (6)	
Mo2	0.794884 (11)	−0.129710 (10)	0.869804 (6)	0.02901 (6)	
N1	0.16496 (13)	0.16445 (10)	0.81190 (7)	0.0292 (3)	
N2	0.17682 (14)	0.07280 (11)	0.92494 (7)	0.0306 (3)	
N3	0.65943 (13)	−0.20844 (10)	0.80556 (7)	0.0302 (3)	
N4	0.67046 (14)	−0.12383 (11)	0.92171 (8)	0.0328 (4)	
O1	0.20034 (11)	−0.01417 (9)	0.82432 (6)	0.0321 (3)	
O2	0.35171 (11)	0.08671 (10)	0.79643 (6)	0.0345 (3)	
O3	0.37760 (13)	0.00636 (11)	0.91758 (7)	0.0426 (3)	
O4	0.34222 (13)	0.17771 (11)	0.90720 (7)	0.0438 (4)	
O5	0.84832 (11)	−0.13193 (10)	0.79391 (7)	0.0349 (3)	
O6	0.69758 (11)	−0.03127 (9)	0.82394 (6)	0.0334 (3)	
O7	0.83513 (13)	−0.22723 (10)	0.90255 (7)	0.0438 (4)	
O8	0.87368 (12)	−0.05671 (10)	0.91652 (7)	0.0399 (3)	

### Atomic displacement parameters (Å^2^)

**Table:**

	*U*^11^	*U*^22^	*U*^33^	*U*^12^	*U*^13^	*U*^23^
C1	0.0297 (9)	0.0297 (9)	0.0318 (10)	−0.0012 (7)	0.0024 (8)	0.0012 (7)
C2	0.0362 (10)	0.0337 (10)	0.0331 (10)	−0.0008 (8)	0.0056 (8)	−0.0025 (8)
C3	0.0444 (12)	0.0312 (10)	0.0472 (12)	−0.0033 (9)	0.0089 (10)	−0.0030 (9)
C4	0.0442 (12)	0.0318 (10)	0.0483 (12)	−0.0044 (9)	0.0106 (10)	0.0072 (9)
C5	0.0479 (12)	0.0362 (11)	0.0386 (11)	−0.0040 (9)	0.0150 (9)	0.0054 (9)
C6	0.0367 (10)	0.0306 (10)	0.0340 (10)	−0.0015 (8)	0.0082 (8)	0.0021 (8)
C7	0.0402 (11)	0.0374 (10)	0.0264 (9)	−0.0009 (8)	0.0080 (8)	0.0007 (8)
C8	0.0441 (11)	0.0333 (10)	0.0277 (9)	0.0003 (8)	0.0103 (8)	−0.0050 (8)
C9	0.0398 (11)	0.0323 (10)	0.0320 (10)	0.0034 (8)	0.0118 (8)	−0.0016 (8)
C10	0.0410 (11)	0.0273 (9)	0.0344 (10)	0.0025 (8)	0.0116 (8)	0.0004 (7)
C11	0.0315 (9)	0.0309 (9)	0.0302 (9)	0.0000 (7)	0.0064 (7)	0.0037 (7)
C12	0.0323 (9)	0.0309 (9)	0.0284 (9)	−0.0039 (8)	0.0084 (7)	0.0009 (7)
C13	0.0353 (10)	0.0399 (11)	0.0309 (10)	−0.0010 (8)	0.0031 (8)	0.0004 (8)
C14	0.0478 (12)	0.0462 (12)	0.0305 (10)	−0.0037 (10)	0.0056 (9)	−0.0080 (9)
C15	0.0488 (12)	0.0396 (11)	0.0370 (11)	0.0020 (9)	0.0133 (9)	−0.0060 (9)
C16	0.0367 (10)	0.0344 (10)	0.0370 (10)	0.0014 (8)	0.0110 (8)	0.0028 (8)
C17	0.0317 (9)	0.0318 (9)	0.0287 (9)	−0.0046 (8)	0.0082 (7)	0.0028 (7)
C18	0.0760 (18)	0.0450 (13)	0.0497 (14)	0.0188 (12)	0.0286 (13)	−0.0016 (11)
C19	0.0382 (12)	0.0511 (13)	0.0502 (13)	−0.0033 (10)	0.0096 (10)	0.0049 (10)
C20	0.0328 (10)	0.0323 (9)	0.0290 (9)	0.0049 (8)	0.0080 (8)	−0.0032 (7)
C21	0.0386 (11)	0.0360 (10)	0.0366 (10)	−0.0023 (8)	0.0118 (9)	−0.0039 (8)
C22	0.0535 (13)	0.0401 (11)	0.0350 (11)	−0.0023 (10)	0.0152 (10)	0.0007 (9)
C23	0.0523 (13)	0.0499 (13)	0.0271 (10)	0.0042 (10)	0.0072 (9)	0.0008 (9)
C24	0.0373 (11)	0.0462 (12)	0.0295 (10)	0.0012 (9)	0.0040 (8)	−0.0059 (8)
C25	0.0333 (10)	0.0348 (10)	0.0284 (9)	0.0027 (8)	0.0078 (8)	−0.0035 (8)
C26	0.0307 (9)	0.0353 (10)	0.0309 (9)	−0.0013 (8)	0.0055 (8)	−0.0064 (8)
C27	0.0395 (11)	0.0303 (9)	0.0336 (10)	−0.0044 (8)	0.0087 (8)	−0.0032 (8)
C28	0.0385 (11)	0.0383 (10)	0.0311 (10)	−0.0057 (8)	0.0103 (8)	−0.0004 (8)
C29	0.0447 (11)	0.0409 (11)	0.0271 (9)	−0.0057 (9)	0.0077 (8)	0.0014 (8)
C30	0.0370 (10)	0.0431 (11)	0.0255 (9)	−0.0003 (9)	0.0063 (8)	−0.0041 (8)
C31	0.0346 (10)	0.0356 (10)	0.0338 (10)	0.0030 (8)	0.0043 (8)	−0.0045 (8)
C32	0.0417 (11)	0.0458 (12)	0.0375 (11)	0.0069 (10)	0.0089 (9)	−0.0110 (9)
C33	0.0451 (12)	0.0380 (11)	0.0512 (13)	0.0072 (9)	0.0049 (10)	−0.0136 (10)
C34	0.0456 (12)	0.0335 (11)	0.0489 (13)	0.0050 (9)	0.0041 (10)	−0.0013 (9)
C35	0.0365 (11)	0.0362 (10)	0.0340 (10)	0.0018 (8)	0.0019 (8)	−0.0002 (8)
C36	0.0304 (9)	0.0330 (10)	0.0289 (9)	0.0026 (7)	0.0002 (7)	−0.0039 (8)
C38	0.0687 (16)	0.0512 (14)	0.0424 (13)	−0.0226 (12)	0.0181 (12)	0.0013 (10)
C37	0.0374 (11)	0.0546 (14)	0.0461 (12)	0.0021 (10)	0.0071 (9)	−0.0067 (11)
Cl1	0.0667 (4)	0.0424 (3)	0.0350 (3)	−0.0004 (2)	0.0116 (2)	−0.0073 (2)
Cl2	0.0819 (4)	0.0338 (3)	0.0620 (4)	−0.0099 (3)	0.0235 (3)	0.0082 (2)
Cl3	0.0450 (3)	0.0685 (4)	0.0535 (3)	0.0216 (3)	0.0118 (3)	0.0018 (3)
Cl4	0.0759 (5)	0.1024 (6)	0.0406 (3)	0.0152 (4)	−0.0076 (3)	−0.0307 (4)
Cl5	0.0453 (3)	0.0663 (4)	0.0554 (3)	−0.0197 (3)	0.0098 (3)	0.0007 (3)
Cl6	0.0823 (5)	0.0978 (6)	0.0345 (3)	−0.0080 (4)	−0.0035 (3)	0.0180 (3)
Cl7	0.0658 (4)	0.0432 (3)	0.0377 (3)	0.0011 (2)	0.0105 (2)	0.0072 (2)
Cl8	0.0860 (5)	0.0460 (3)	0.0697 (4)	0.0152 (3)	0.0148 (4)	−0.0221 (3)
Mo1	0.02827 (9)	0.03244 (9)	0.02461 (9)	−0.00161 (5)	0.00267 (6)	−0.00068 (5)
Mo2	0.02898 (9)	0.03137 (9)	0.02493 (9)	0.00155 (5)	0.00191 (6)	−0.00017 (5)
N1	0.0310 (8)	0.0272 (8)	0.0301 (8)	−0.0017 (6)	0.0082 (6)	0.0002 (6)
N2	0.0367 (9)	0.0304 (8)	0.0238 (8)	−0.0002 (6)	0.0044 (6)	−0.0016 (6)
N3	0.0315 (8)	0.0292 (8)	0.0301 (8)	0.0013 (6)	0.0071 (6)	−0.0024 (6)
N4	0.0357 (9)	0.0368 (9)	0.0245 (8)	−0.0019 (7)	0.0030 (7)	−0.0010 (6)
O1	0.0389 (7)	0.0301 (7)	0.0269 (6)	−0.0051 (6)	0.0060 (5)	0.0003 (5)
O2	0.0287 (7)	0.0453 (8)	0.0292 (7)	−0.0027 (6)	0.0056 (5)	0.0006 (6)
O3	0.0407 (8)	0.0527 (9)	0.0323 (7)	0.0086 (7)	0.0031 (6)	0.0048 (7)
O4	0.0448 (9)	0.0439 (8)	0.0407 (8)	−0.0096 (7)	0.0044 (7)	−0.0090 (7)
O5	0.0291 (7)	0.0444 (8)	0.0305 (7)	0.0035 (6)	0.0050 (6)	−0.0004 (6)
O6	0.0392 (7)	0.0331 (7)	0.0264 (6)	0.0071 (6)	0.0040 (5)	−0.0011 (5)
O7	0.0459 (9)	0.0393 (8)	0.0430 (8)	0.0063 (7)	0.0021 (7)	0.0051 (7)
O8	0.0409 (8)	0.0456 (8)	0.0307 (7)	−0.0071 (7)	0.0020 (6)	−0.0024 (6)

### Geometric parameters (Å, º)

**Table:**

C1—O1	1.302 (2)	C22—H22A	0.9300
C1—C6	1.413 (3)	C23—C24	1.378 (3)
C1—C2	1.414 (3)	C23—Cl6	1.736 (2)
C2—C3	1.373 (3)	C24—C25	1.395 (3)
C2—Cl1	1.727 (2)	C24—H24A	0.9300
C3—C4	1.397 (3)	C25—C26	1.461 (3)
C3—H3A	0.9300	C26—N3	1.271 (3)
C4—C5	1.373 (3)	C26—H26A	0.9300
C4—Cl2	1.735 (2)	C27—N3	1.472 (3)
C5—C6	1.408 (3)	C27—C28	1.535 (3)
C5—H5A	0.9300	C27—H27A	0.9700
C6—C7	1.443 (3)	C27—H27B	0.9700
C7—N2	1.291 (3)	C28—C37	1.522 (3)
C7—H7A	0.9300	C28—C38	1.533 (3)
C8—N2	1.470 (3)	C28—C29	1.539 (3)
C8—C9	1.540 (3)	C29—N4	1.475 (3)
C8—H8A	0.9700	C29—H29A	0.9700
C8—H8B	0.9700	C29—H29B	0.9700
C9—C19	1.524 (3)	C30—N4	1.293 (3)
C9—C10	1.534 (3)	C30—C31	1.443 (3)
C9—C18	1.538 (3)	C30—H30A	0.9300
C10—N1	1.473 (2)	C31—C32	1.408 (3)
C10—H10A	0.9700	C31—C36	1.419 (3)
C10—H10B	0.9700	C32—C33	1.371 (4)
C11—N1	1.275 (3)	C32—H32A	0.9300
C11—C12	1.460 (3)	C33—C34	1.389 (4)
C11—H11A	0.9300	C33—Cl8	1.737 (2)
C12—C13	1.392 (3)	C34—C35	1.376 (3)
C12—C17	1.403 (3)	C34—H34A	0.9300
C13—C14	1.379 (3)	C35—C36	1.414 (3)
C13—H13A	0.9300	C35—Cl7	1.725 (2)
C14—C15	1.381 (3)	C36—O6	1.301 (2)
C14—Cl4	1.734 (2)	C38—H38A	0.9600
C15—C16	1.381 (3)	C38—H38B	0.9600
C15—H15A	0.9300	C38—H38C	0.9600
C16—C17	1.396 (3)	C37—H37A	0.9600
C16—Cl3	1.724 (2)	C37—H37B	0.9600
C17—O2	1.343 (2)	C37—H37C	0.9600
C18—H18A	0.9600	Mo1—O4	1.7068 (16)
C18—H18B	0.9600	Mo1—O3	1.7075 (16)
C18—H18C	0.9600	Mo1—O2	1.9467 (15)
C19—H19A	0.9600	Mo1—O1	2.0931 (14)
C19—H19B	0.9600	Mo1—N2	2.1547 (18)
C19—H19C	0.9600	Mo1—N1	2.3347 (17)
C20—O5	1.339 (2)	Mo2—O7	1.7045 (16)
C20—C21	1.398 (3)	Mo2—O8	1.7088 (15)
C20—C25	1.404 (3)	Mo2—O5	1.9493 (15)
C21—C22	1.383 (3)	Mo2—O6	2.0893 (14)
C21—Cl5	1.726 (2)	Mo2—N4	2.1621 (18)
C22—C23	1.383 (3)	Mo2—N3	2.3391 (17)
			
O1—C1—C6	122.22 (18)	C28—C27—H27A	108.4
O1—C1—C2	120.33 (18)	N3—C27—H27B	108.4
C6—C1—C2	117.41 (18)	C28—C27—H27B	108.4
C3—C2—C1	121.8 (2)	H27A—C27—H27B	107.5
C3—C2—Cl1	120.50 (17)	C37—C28—C38	110.3 (2)
C1—C2—Cl1	117.70 (16)	C37—C28—C27	111.21 (18)
C2—C3—C4	119.4 (2)	C38—C28—C27	105.49 (18)
C2—C3—H3A	120.3	C37—C28—C29	111.15 (19)
C4—C3—H3A	120.3	C38—C28—C29	107.14 (18)
C5—C4—C3	120.9 (2)	C27—C28—C29	111.36 (18)
C5—C4—Cl2	120.20 (18)	N4—C29—C28	112.10 (16)
C3—C4—Cl2	118.92 (17)	N4—C29—H29A	109.2
C4—C5—C6	119.8 (2)	C28—C29—H29A	109.2
C4—C5—H5A	120.1	N4—C29—H29B	109.2
C6—C5—H5A	120.1	C28—C29—H29B	109.2
C5—C6—C1	120.35 (19)	H29A—C29—H29B	107.9
C5—C6—C7	119.94 (19)	N4—C30—C31	125.29 (19)
C1—C6—C7	119.09 (18)	N4—C30—H30A	117.4
N2—C7—C6	125.10 (19)	C31—C30—H30A	117.4
N2—C7—H7A	117.4	C32—C31—C36	120.5 (2)
C6—C7—H7A	117.4	C32—C31—C30	120.0 (2)
N2—C8—C9	112.39 (16)	C36—C31—C30	118.80 (18)
N2—C8—H8A	109.1	C33—C32—C31	119.5 (2)
C9—C8—H8A	109.1	C33—C32—H32A	120.2
N2—C8—H8B	109.1	C31—C32—H32A	120.2
C9—C8—H8B	109.1	C32—C33—C34	121.3 (2)
H8A—C8—H8B	107.9	C32—C33—Cl8	120.28 (19)
C19—C9—C10	110.89 (18)	C34—C33—Cl8	118.42 (19)
C19—C9—C18	110.3 (2)	C35—C34—C33	119.6 (2)
C10—C9—C18	106.04 (18)	C35—C34—H34A	120.2
C19—C9—C8	110.93 (18)	C33—C34—H34A	120.2
C10—C9—C8	111.76 (17)	C34—C35—C36	121.6 (2)
C18—C9—C8	106.77 (18)	C34—C35—Cl7	120.93 (18)
N1—C10—C9	115.33 (16)	C36—C35—Cl7	117.48 (16)
N1—C10—H10A	108.4	O6—C36—C35	120.32 (18)
C9—C10—H10A	108.4	O6—C36—C31	122.35 (18)
N1—C10—H10B	108.4	C35—C36—C31	117.29 (18)
C9—C10—H10B	108.4	C28—C38—H38A	109.5
H10A—C10—H10B	107.5	C28—C38—H38B	109.5
N1—C11—C12	124.97 (18)	H38A—C38—H38B	109.5
N1—C11—H11A	117.5	C28—C38—H38C	109.5
C12—C11—H11A	117.5	H38A—C38—H38C	109.5
C13—C12—C17	120.04 (18)	H38B—C38—H38C	109.5
C13—C12—C11	117.89 (18)	C28—C37—H37A	109.5
C17—C12—C11	122.03 (18)	C28—C37—H37B	109.5
C14—C13—C12	119.9 (2)	H37A—C37—H37B	109.5
C14—C13—H13A	120.1	C28—C37—H37C	109.5
C12—C13—H13A	120.1	H37A—C37—H37C	109.5
C13—C14—C15	121.1 (2)	H37B—C37—H37C	109.5
C13—C14—Cl4	119.27 (18)	O4—Mo1—O3	103.91 (8)
C15—C14—Cl4	119.58 (17)	O4—Mo1—O2	102.62 (7)
C16—C15—C14	118.9 (2)	O3—Mo1—O2	105.53 (7)
C16—C15—H15A	120.5	O4—Mo1—O1	161.13 (7)
C14—C15—H15A	120.5	O3—Mo1—O1	91.92 (8)
C15—C16—C17	121.6 (2)	O2—Mo1—O1	82.50 (6)
C15—C16—Cl3	119.61 (17)	O4—Mo1—N2	90.76 (7)
C17—C16—Cl3	118.72 (16)	O3—Mo1—N2	92.73 (7)
O2—C17—C16	119.67 (18)	O2—Mo1—N2	153.75 (6)
O2—C17—C12	121.91 (18)	O1—Mo1—N2	78.11 (6)
C16—C17—C12	118.33 (18)	O4—Mo1—N1	84.66 (7)
C9—C18—H18A	109.5	O3—Mo1—N1	168.18 (7)
C9—C18—H18B	109.5	O2—Mo1—N1	80.02 (6)
H18A—C18—H18B	109.5	O1—Mo1—N1	78.31 (6)
C9—C18—H18C	109.5	N2—Mo1—N1	78.86 (6)
H18A—C18—H18C	109.5	O7—Mo2—O8	103.88 (8)
H18B—C18—H18C	109.5	O7—Mo2—O5	102.80 (7)
C9—C19—H19A	109.5	O8—Mo2—O5	105.23 (7)
C9—C19—H19B	109.5	O7—Mo2—O6	161.15 (7)
H19A—C19—H19B	109.5	O8—Mo2—O6	91.63 (7)
C9—C19—H19C	109.5	O5—Mo2—O6	82.94 (6)
H19A—C19—H19C	109.5	O7—Mo2—N4	90.08 (7)
H19B—C19—H19C	109.5	O8—Mo2—N4	93.36 (7)
O5—C20—C21	119.83 (19)	O5—Mo2—N4	153.84 (6)
O5—C20—C25	122.10 (18)	O6—Mo2—N4	78.20 (6)
C21—C20—C25	118.01 (19)	O7—Mo2—N3	85.25 (7)
C22—C21—C20	121.8 (2)	O8—Mo2—N3	168.18 (7)
C22—C21—Cl5	119.51 (17)	O5—Mo2—N3	79.55 (6)
C20—C21—Cl5	118.73 (17)	O6—Mo2—N3	78.10 (6)
C23—C22—C21	119.0 (2)	N4—Mo2—N3	78.94 (6)
C23—C22—H22A	120.5	C11—N1—C10	118.13 (17)
C21—C22—H22A	120.5	C11—N1—Mo1	122.73 (13)
C24—C23—C22	121.2 (2)	C10—N1—Mo1	118.80 (12)
C24—C23—Cl6	119.62 (19)	C7—N2—C8	118.14 (18)
C22—C23—Cl6	119.22 (18)	C7—N2—Mo1	122.83 (14)
C23—C24—C25	119.8 (2)	C8—N2—Mo1	119.01 (13)
C23—C24—H24A	120.1	C26—N3—C27	117.66 (17)
C25—C24—H24A	120.1	C26—N3—Mo2	123.15 (14)
C24—C25—C20	120.29 (19)	C27—N3—Mo2	118.86 (12)
C24—C25—C26	118.05 (19)	C30—N4—C29	118.01 (18)
C20—C25—C26	121.63 (18)	C30—N4—Mo2	122.70 (15)
N3—C26—C25	124.80 (18)	C29—N4—Mo2	119.22 (13)
N3—C26—H26A	117.6	C1—O1—Mo1	122.11 (12)
C25—C26—H26A	117.6	C17—O2—Mo1	126.50 (12)
N3—C27—C28	115.55 (16)	C20—O5—Mo2	126.63 (12)
N3—C27—H27A	108.4	C36—O6—Mo2	121.74 (12)
			
O1—C1—C2—C3	175.4 (2)	C12—C11—N1—C10	173.77 (18)
C6—C1—C2—C3	−6.7 (3)	C12—C11—N1—Mo1	0.5 (3)
O1—C1—C2—Cl1	−2.2 (3)	C9—C10—N1—C11	126.2 (2)
C6—C1—C2—Cl1	175.74 (15)	C9—C10—N1—Mo1	−60.2 (2)
C1—C2—C3—C4	3.2 (3)	O4—Mo1—N1—C11	129.88 (16)
Cl1—C2—C3—C4	−179.30 (18)	O3—Mo1—N1—C11	−93.0 (3)
C2—C3—C4—C5	2.5 (4)	O2—Mo1—N1—C11	26.04 (15)
C2—C3—C4—Cl2	−177.56 (18)	O1—Mo1—N1—C11	−58.29 (15)
C3—C4—C5—C6	−4.5 (4)	N2—Mo1—N1—C11	−138.29 (16)
Cl2—C4—C5—C6	175.64 (18)	O4—Mo1—N1—C10	−43.37 (14)
C4—C5—C6—C1	0.7 (3)	O3—Mo1—N1—C10	93.7 (3)
C4—C5—C6—C7	171.6 (2)	O2—Mo1—N1—C10	−147.21 (14)
O1—C1—C6—C5	−177.4 (2)	O1—Mo1—N1—C10	128.47 (14)
C2—C1—C6—C5	4.7 (3)	N2—Mo1—N1—C10	48.47 (13)
O1—C1—C6—C7	11.6 (3)	C6—C7—N2—C8	164.3 (2)
C2—C1—C6—C7	−166.30 (19)	C6—C7—N2—Mo1	−14.2 (3)
C5—C6—C7—N2	164.3 (2)	C9—C8—N2—C7	−102.7 (2)
C1—C6—C7—N2	−24.7 (3)	C9—C8—N2—Mo1	75.8 (2)
N2—C8—C9—C19	58.3 (2)	O4—Mo1—N2—C7	−152.81 (17)
N2—C8—C9—C10	−66.0 (2)	O3—Mo1—N2—C7	−48.85 (17)
N2—C8—C9—C18	178.51 (19)	O2—Mo1—N2—C7	85.8 (2)
C19—C9—C10—N1	−64.0 (2)	O1—Mo1—N2—C7	42.54 (16)
C18—C9—C10—N1	176.33 (19)	N1—Mo1—N2—C7	122.78 (17)
C8—C9—C10—N1	60.4 (2)	O4—Mo1—N2—C8	28.72 (15)
N1—C11—C12—C13	162.51 (19)	O3—Mo1—N2—C8	132.69 (15)
N1—C11—C12—C17	−19.9 (3)	O2—Mo1—N2—C8	−92.67 (19)
C17—C12—C13—C14	2.2 (3)	O1—Mo1—N2—C8	−135.92 (15)
C11—C12—C13—C14	179.79 (19)	N1—Mo1—N2—C8	−55.69 (14)
C12—C13—C14—C15	−1.1 (3)	C25—C26—N3—C27	−174.48 (18)
C12—C13—C14—Cl4	179.31 (17)	C25—C26—N3—Mo2	−1.1 (3)
C13—C14—C15—C16	−0.6 (4)	C28—C27—N3—C26	−126.1 (2)
Cl4—C14—C15—C16	178.99 (18)	C28—C27—N3—Mo2	60.2 (2)
C14—C15—C16—C17	1.2 (3)	O7—Mo2—N3—C26	−129.83 (17)
C14—C15—C16—Cl3	−176.71 (18)	O8—Mo2—N3—C26	89.1 (3)
C15—C16—C17—O2	−176.79 (19)	O5—Mo2—N3—C26	−25.86 (16)
Cl3—C16—C17—O2	1.2 (3)	O6—Mo2—N3—C26	59.05 (16)
C15—C16—C17—C12	−0.2 (3)	N4—Mo2—N3—C26	139.16 (17)
Cl3—C16—C17—C12	177.76 (15)	O7—Mo2—N3—C27	43.50 (14)
C13—C12—C17—O2	175.00 (18)	O8—Mo2—N3—C27	−97.5 (3)
C11—C12—C17—O2	−2.5 (3)	O5—Mo2—N3—C27	147.47 (14)
C13—C12—C17—C16	−1.5 (3)	O6—Mo2—N3—C27	−127.63 (14)
C11—C12—C17—C16	−179.01 (18)	N4—Mo2—N3—C27	−47.51 (13)
O5—C20—C21—C22	178.03 (19)	C31—C30—N4—C29	−164.04 (19)
C25—C20—C21—C22	0.7 (3)	C31—C30—N4—Mo2	12.8 (3)
O5—C20—C21—Cl5	−1.5 (3)	C28—C29—N4—C30	101.2 (2)
C25—C20—C21—Cl5	−178.87 (15)	C28—C29—N4—Mo2	−75.8 (2)
C20—C21—C22—C23	−1.3 (3)	O7—Mo2—N4—C30	152.99 (17)
Cl5—C21—C22—C23	178.23 (18)	O8—Mo2—N4—C30	49.08 (17)
C21—C22—C23—C24	−0.1 (4)	O5—Mo2—N4—C30	−86.7 (2)
C21—C22—C23—Cl6	178.85 (18)	O6—Mo2—N4—C30	−41.89 (16)
C22—C23—C24—C25	2.1 (3)	N3—Mo2—N4—C30	−121.87 (17)
Cl6—C23—C24—C25	−176.87 (17)	O7—Mo2—N4—C29	−30.24 (15)
C23—C24—C25—C20	−2.7 (3)	O8—Mo2—N4—C29	−134.14 (15)
C23—C24—C25—C26	179.1 (2)	O5—Mo2—N4—C29	90.1 (2)
O5—C20—C25—C24	−175.97 (18)	O6—Mo2—N4—C29	134.89 (15)
C21—C20—C25—C24	1.3 (3)	N3—Mo2—N4—C29	54.90 (14)
O5—C20—C25—C26	2.2 (3)	C6—C1—O1—Mo1	39.7 (3)
C21—C20—C25—C26	179.52 (18)	C2—C1—O1—Mo1	−142.49 (16)
C24—C25—C26—N3	−161.2 (2)	O4—Mo1—O1—C1	−110.6 (2)
C20—C25—C26—N3	20.6 (3)	O3—Mo1—O1—C1	36.71 (15)
N3—C27—C28—C37	63.1 (2)	O2—Mo1—O1—C1	142.12 (15)
N3—C27—C28—C38	−177.35 (19)	N2—Mo1—O1—C1	−55.68 (15)
N3—C27—C28—C29	−61.5 (2)	N1—Mo1—O1—C1	−136.58 (15)
C37—C28—C29—N4	−57.7 (2)	C16—C17—O2—Mo1	−133.78 (16)
C38—C28—C29—N4	−178.25 (19)	C12—C17—O2—Mo1	49.8 (2)
C27—C28—C29—N4	66.9 (2)	O4—Mo1—O2—C17	−132.81 (16)
N4—C30—C31—C32	−164.6 (2)	O3—Mo1—O2—C17	118.66 (16)
N4—C30—C31—C36	25.2 (3)	O1—Mo1—O2—C17	28.74 (16)
C36—C31—C32—C33	−1.4 (3)	N2—Mo1—O2—C17	−13.8 (2)
C30—C31—C32—C33	−171.5 (2)	N1—Mo1—O2—C17	−50.63 (16)
C31—C32—C33—C34	4.1 (4)	C21—C20—O5—Mo2	132.65 (17)
C31—C32—C33—Cl8	−176.35 (18)	C25—C20—O5—Mo2	−50.1 (3)
C32—C33—C34—C35	−2.1 (4)	O7—Mo2—O5—C20	133.49 (16)
Cl8—C33—C34—C35	178.43 (18)	O8—Mo2—O5—C20	−118.05 (17)
C33—C34—C35—C36	−2.8 (3)	O6—Mo2—O5—C20	−28.27 (16)
C33—C34—C35—Cl7	179.34 (18)	N4—Mo2—O5—C20	15.7 (3)
C34—C35—C36—O6	−177.0 (2)	N3—Mo2—O5—C20	50.86 (16)
Cl7—C35—C36—O6	0.9 (3)	C35—C36—O6—Mo2	141.28 (16)
C34—C35—C36—C31	5.3 (3)	C31—C36—O6—Mo2	−41.2 (2)
Cl7—C35—C36—C31	−176.78 (15)	O7—Mo2—O6—C36	108.9 (2)
C32—C31—C36—O6	179.22 (19)	O8—Mo2—O6—C36	−36.78 (16)
C30—C31—C36—O6	−10.6 (3)	O5—Mo2—O6—C36	−141.92 (16)
C32—C31—C36—C35	−3.2 (3)	N4—Mo2—O6—C36	56.31 (15)
C30—C31—C36—C35	167.01 (19)	N3—Mo2—O6—C36	137.32 (16)

### Hydrogen-bond geometry (Å, º)

*Cg1* is the centroid of the C20–C25 ring in molecule *B* and *Cg2* is the centroid of the C12–C17 ring in molecule *A*.

**Table:**

*D*—H···*A*	*D*—H	H···*A*	*D*···*A*	*D*—H···*A*
C38—H38*B*···Cl8^i^	0.96	2.82	3.767 (3)	168
C10—H10*B*···*Cg*1^ii^	0.97	2.66	3.433 (3)	136
C27—H27*A*···*Cg*2^iii^	0.97	2.55	3.363 (3)	141

Symmetry codes: (i) −*x*+1, −*y*, −*z*+2; (ii) −*x*+1, *y*+1/2, −*z*+3/2; (iii) −*x*+1, *y*−1/2, −*z*+3/2.
